# Insights Into the Changing Landscape of Coronavirus Disease 2019

**DOI:** 10.3389/fcimb.2021.761521

**Published:** 2022-01-10

**Authors:** Ruby A. Escobedo, Deepak Kaushal, Dhiraj K. Singh

**Affiliations:** ^1^ Southwest National Primate Research Center, Texas Biomedical Research Institute, San Antonio, TX, United States; ^2^ The Integrated Biomedical Sciences (IBMS) Graduate Program, University of Texas Health Sciences Center at San Antonio, San Antonio, TX, United States

**Keywords:** COVID-19, SARS-CoV-2, coronavirus, targeted therapy, COVID treatment, COVID origin, COVID epidemiology

## Abstract

Coronavirus disease 2019 (COVID-19) is a highly contagious, infectious disease caused by severe acute respiratory syndrome coronavirus 2 (SARS-CoV-2), which emerged in late 2019 in Wuhan China. A year after the World Health Organization declared COVID-19 a global pandemic, over 215 million confirmed cases and approximately 5 million deaths have been reported worldwide. In this multidisciplinary review, we summarize important insights for COVID-19, ranging from its origin, pathology, epidemiology, to clinical manifestations and treatment. More importantly, we also highlight the foundational connection between genetics and the development of personalized medicine and how these aspects have an impact on disease treatment and management in the dynamic landscape of this pandemic.

## Introduction

The threat to human health posed by coronaviruses (CoV) has recently been exemplified by three highly contagious CoVs that have spread rapidly throughout the world in the past two decades. In 2002, a novel Coronavirus, termed severe acute respiratory syndrome associated coronavirus (SARS-CoV), appeared in Guangdong Province, China, and affected 8,098 people, and resulted in 774 deaths in 26 countries (Cherry and Krogstad, 2004; Leduc and Barry, 2004). SARS-CoV was later deemed the causative agent of the severe acute respiratory syndrome (SARS). Ten years after the SARS-CoV epidemic, the first cases of Middle East Respiratory Syndrome (MERS) were reported in Saudi Arabia and the causative agent was again identified as a novel CoV named MERS-CoV. MERS-CoV affected 2,519 people and caused 866 deaths in 27 countries (De Wit et al., 2016). Late in December of 2019, reports of pneumonia with unknown etiology appeared in Wuhan, China. The pneumonia was later determined to be caused by another novel CoV, named SARS-CoV-2, for its genetic similarities to SARS-CoV. As of November 2021, over 215 million confirmed cases and approximately 5 million deaths had been recorded in 222 countries and territories. SARS-CoV-2 has turned into a global pandemic and is a substantial threat to human health (WHO, 2020b).

On January 30 2020, the World Health Organization (WHO) declared coronavirus disease 2019 (COVID-19) caused by SARS-CoV-2, a Public Health Emergency of International Concern. By the time WHO declared the COVID-19 outbreak a pandemic, on March 11, 2020, more than 100,000 people in at least 114 countries were already affected (WHO, 2020b). While one-fifth of COVID-19 patients remained asymptomatic, many developed mild symptoms such as dry cough, sore throat, and fever (Kim et al., 2020). A small percentage of the infected population develop fatal complications including organ failure, septic shock, pulmonary edema, severe pneumonia, and acute respiratory distress syndrome (ARDS). Those in intensive care were also most likely to report dyspnea, dizziness, abdominal pain, and anorexia (Sohrabi et al., 2020). A majority of hospitalized patients exhibiting severe symptoms were either from an elderly age group or possessed preexisting health conditions.

This review covers the important aspects of what is currently known about SARS-CoV-2, with a focus on the identification of genetic markers to susceptibility or resistance to infection. This section also considers varied innate immune responses that correspond to a variety of clinical manifestations.

## Origin of SARS-CoV-2

**Figure 1:**
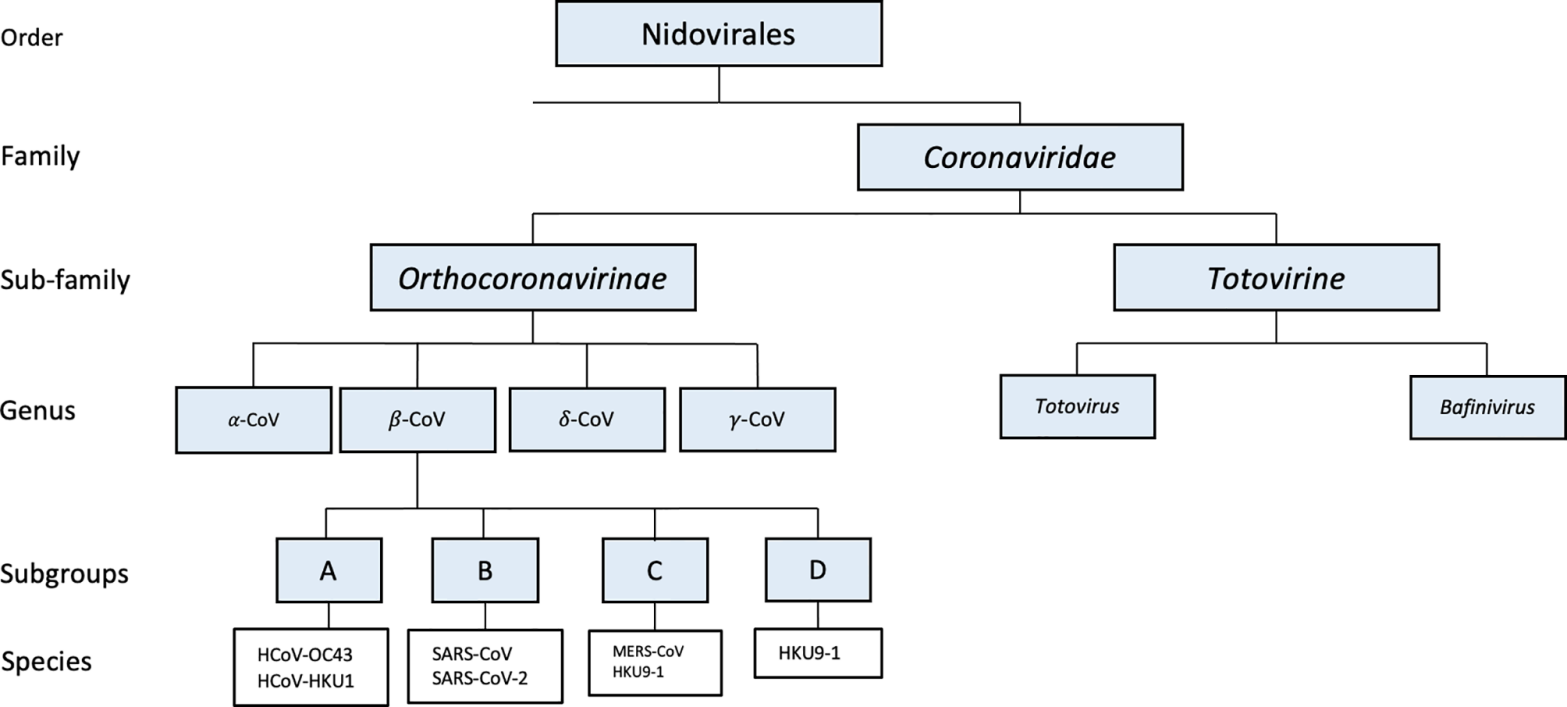
Phylogenetic tree of SARS-CoV-2 and other related coronaviruses.

Identifying the origin of an emerging pathogen is critical for the prevention and control of future outbreaks. Based on phylogenetic and taxonomic studies, 2019 novel CoV (2019-nCoV) was identified as forming a new sister clade to the prototype human and bat SARS-CoVs of the SARS-CoV species (Gorbalenya et al., 2020). With approximately 79% genetic similarity to SARS-CoV and its disease resemblance to SARS-CoV, 2019-CoV pathogen was designated as Severe Acute Respiratory Syndrome associated Coronavirus 2 (SARS-CoV-2) (Lu et al., 2020). The genetic homology between β-CoVs found to infect bats and SARS-CoV-2 suggests that this novel CoV may have evolved from CoVs that are known to infect bats. SARS-CoV-2, like SARS-CoV and MERS-CoV, is a β-CoV that belongs to the family Coronaviridae within the Nidovirales order (Woo et al., 2010). The subfamily Orthocoronavirinae is further divided into four genera: alpha, beta, delta, and gamma. There are nearly 30 characterized CoV’s that infect humans, mammals, fowl, and a wide array of other animals (Li et al., 2005). Genomic classification of CoVs often correlates to which host said CoV will infect. For example, while delta- and gamma-CoVs typically infect birds, alpha and beta CoVs are known to infect mammals, and are also further classified into four different lineages (A-D) (**Figure 1**) (Cui et al., 2019; Guo et al., 2020; Wu et al., 2020; Zhou et al., 2020).

Genetic analyses have shed light on the similarities of SARS-CoV-2 to other SARS-like CoVs. To properly classify SARS-CoV-2, the viral genome was sequenced and compared to other prominent CoVs. Notably, SARS-CoV-2 displays greater conservation to bat CoV RaTG13 (96.2%) than SARS-CoV (approximately 79%) and MERS-CoV (approximately 50%) (Malaiyan et al., 2020; Zhou et al., 2020). It was determined that SARS-CoV-2 utilizes the same host receptor, angiotensin-converting enzyme 2 (ACE2), which SARS-CoV also utilizes for cell entry (Tang et al., 2020; Zhou et al., 2020). The viruses gain entry to the host cell *via* the receptor-binding domain (RBD) on the spike (S) protein. While both viruses use the same host receptor to gain entry, RBD of S protein on SARS-CoV-2 has a binding affinity 20 times higher to ACE2 compared to the S protein on SARS-CoV (Guo et al., 2020; Wrapp et al., 2020; Zhou et al., 2020). Other studies have found SARS-CoV to have a lower binding affinity to ACE2 when compared to SARS-CoV-2 (with dissociation constants of 31 nM compared to 4.7 nM, respectively) (Lan et al., 2020). Another striking feature that SARS-CoV-2 utilizes for enhanced cell entry is the pre-activation of the S protein by the proprotein convertase furin rather than host cellular proteases (Shang et al., 2020a). These three factors might collectively impart an essential survival advantage to the novel SARS-CoV-2 in terms of higher and more efficient infectivity and survival in the host while also rendering host immune surveillance less effective.

After the first large-scale epidemic caused by SARS-CoV in 2002, studies have demonstrated that the genetic diversity found in zoonotic viruses in bats increases the possibility of crossing the species barrier (Li et al., 2005). The pathogenic CoVs, SARS-CoV, MERS-CoV, and SARS-CoV-2, have all pointed towards an origin associated with CoVs infecting bats as their natural host reservoir due to the genetic similarities found in SARS-CoV-related viruses in horseshoe bats (genus *Rhinolophus*) (Cui et al., 2019). However, the transmission from bats to humans often involves an intermediate host (Deng et al., 2020; Guo et al., 2020). The intermediate hosts are often found by the detection of antibodies against the virus in animals that show no evidence for the circulation of SARS-and MERS-CoV-like viruses (De Wit et al., 2016). For SARS-CoV and MERS-CoV, the intermediate hosts were discovered to be palm civets and dromedary camels, respectively (Tu et al., 2004; Hemida et al., 2013). With considerable controversy surrounding intermediate hosts for SARS-CoV-2, initial studies suggested the pangolin as the source of SARS-CoV-2 due to the occurrence of genetic and evolutionary evidence found in SARS-CoV-2-like CoVs in pangolins (Liu et al., 2020a; Zhang et al., 2020b). However, this claim was rejected when other studies analyzed ACE2 from Chinese pangolin, Sunda pangolin, and white-bellied pangolin and found a low or very low binding affinity for SARS-CoV-2 RBD (Damas et al., 2020). This claim is also supported by another study that identified unique peptide motifs in SARS-CoV-2 that were absent in CoVs isolated from pangolins, concluding that the human SARS-CoV-2 virus did not come directly from pangolins (Li et al., 2020c). Other studies have also suggested snakes and turtles as probable host intermediates, which were later contradicted by another study that compared ACE2 affinity to SARS-CoV-2 RBD in turtles and snakes concluding neither were intermediate hosts for SARS-CoV-2 (Deng et al., 2020; Luan et al., 2020).

Interestingly, SARS-CoV-2 infection predominantly manifests through respiratory symptoms, however, studies have shown that the overall expression of ACE2 is relatively low in human tissues, especially pulmonary and bronchial tissues (Sungnak et al., 2020). This may suggest that SARS-CoV-2 may use an additional receptor for cell entry (Wang et al., 2021c). On this note, a study observed that the presence of high-density lipoprotein (HDL) scavenger receptor B type 1 (SR-B1) facilitated the ACE2-dependent entry of SARS-CoV-2 (Wei et al., 2020). While SR-B1 alone cannot prevent cell entry, it is thought the mechanism behind this facilitation is that SR-B1 recruits viral particles to ACE2 with HDL. Another study by Wang et al. demonstrated that tyrosine-protein kinase receptor UFO (AXL) found on pulmonary and bronchial cells specifically interacts with SARS-CoV-2 S. When AXL, but not ACE2, was downregulated in pulmonary cells, they found a significant reduction in SARS-CoV-2 infection. These data suggest there may be additional factors for viral attachment to host cell membranes.

Because viruses constantly change through mutation, a variant emerges when one or more mutations occur and is differentiated from other variants in circulation. SARS-CoV-2 viruses have two major clades (L and S), and are defined by just two single nucleotide polymorphisms (SNPs) that show complete linkage across SARS-CoV-2 strains. The L clade (∼70%) was found to be more prevalent than the S clade (∼30%) in the SARS-CoV-2 viruses that were examined. Evolutionary analyses suggest that the S clade appeared to be more related to CoVs in animals (Tang et al., 2020). The genetic analysis reported by GISAID further revealed eight distinct clades based on marker variants including L, S, G, GH, GR, GV, GRY and V (https://www.gisaid.org) (Elbe and Buckland-Merrett, 2017). Because genetic variation/SNPs are main contributors to altered protein translation and function, a study investigated SNPs from geographically distributed SARS-CoV-2 strains and found distinct SNPs within clades- G clade with C241T, C3037T and A23403G, GR with C241T, C3037T, A23403G, and G28882A, GH clade with C241T, C3037T, A23403G and G25563T, S clade with C8782T and T28144C, V clade with G11083T and G26144T (Yuan et al., 2021). Additionally, among the mutations found, nucleotide substitutions from C to T accounted for nearly half of SNPs, reducing the number of CpG sites. CpG sites are essential for zinc-finger anti-viral proteins to bind and perform viral RNA genome degradation (Yuan et al., 2021).

Furthermore, a US government SARS-CoV-2 Interagency Group (SIG) has further devised a schematic for variant classification. According to this group, SARS-CoV-2 variants can be classified into three classes: variant of interest, variant of concern, and variant of high consequence. As of now, there are currently no variants of high consequence circulating in the United States, but there are four variants of concern in circulation: B.1.1.7 (Alpha), B.1.351 (Beta), B.1.617.2 (Delta), and P.1 (Gamma). Considering most vaccines are designed against the SARS-CoV-2 spike protein, mutations within this region are likely to result in less effective vaccinations (Escobedo et al., 2021). However, a study focusing on the effectiveness of the AstraZeneca and Pfizer vaccines against the predominant variant (B.1.1.7) and the delta variant surging through India and the United Kingdom (B.1.617.2) showed small differences between both vaccines, suggesting that both vaccines may continue to protect against SARS-CoV-2 strains (Lopez Bernal et al., 2021).

## Virology/Pathology

CoVs are enveloped RNA viruses that are known to possess some of the largest RNA viral genomes (26.4 to 31.7 Kb) (Woo et al., 2010). The genome consists of single-stranded positive-sense RNA (ss+RNA) with a 5′‐cap structure and 3′‐poly-A tail. In addition, the genome contains 10 identified genes and 14 open reading frames (ORFs) encoding 27 proteins (Azkur et al., 2020; Chen et al., 2020b). ORF1a/b comprises two-thirds of the genome at the 5’-terminal and encodes polyproteins 1a/1ab. These polyproteins comprise the major nonstructural proteins (NSPs), which are proteins that are not involved in virion formation. Specific ORF1a-encoded transmembrane domains that reside in nsp3, nsp4, and nsp6 have been implicated to form the replication-transcription complex (RTC) in a double-membrane vesicle (DMV), a phenomenon necessary for viral replication with nsp13 driving the formation (Snijder et al., 2006; Chen et al., 2020a; Chen et al., 2020b). The remaining third of the genome at the 3’ terminal consists of other ORFs containing the four main structural proteins: spike (S), envelope (E), membrane (M), and nucleocapsid (N) (Chen et al., 2020b; Wu et al., 2020). The most defining structure of all CoVs is the club-like S proteins around the virion, giving the virus the name “coronavirus”, which is derived from Latin *corona*, meaning “crown”.

Similar to other CoVs, the genome of SARS-CoV-2 is 29.9 kb and is composed of a nucleocapsid with genomic RNA and phosphorylated nucleocapsid (N) protein, which is buried inside the phospholipid bilayers (Wu et al., 2020). The outer membrane of the virion consists of S, E, and M proteins. The S protein is critical for host receptor interaction. Interactions of the S protein allow the virus to gain cell entry and propagate further infection. For a successful infection, the virus must bind to the host receptor, followed by cleavage of the S protein and fusion with the cell membrane. SARS-CoV-2 binds to the ACE2 receptor on the host cell for the gain of entry, similar to SARS-CoV and HCoV-NL63.

ACE2 is a type 1 membrane protein that acts as a negative regulator of octapeptide angiotensin II (ANG II) within the renin-angiotensin system (RAS) and is expressed in various cells types (Kuba et al., 2006). This includes type II lung alveolar epithelial cells and enterocytes of the small intestine; arterial and venous endothelial cells, and arterial smooth muscle cells in various organs (Hamming et al., 2004). While ACE2 is expressed in many organs, in the lungs it is mostly expressed by ciliated bronchial epithelial cells and type II pneumocytes, making them the primary targets for initial SARS-CoV-2 infection, during airborne transmission (Fung et al., 2020). Studies have also demonstrated that an increase of surface ACE2 expression may potentially lead to increased susceptibility to infection (Devaux et al., 2020).

The S protein includes two subunits, S1 and S2 (Ou et al., 2020). While S1 determines the host range and facilitates viral attachment to the target cells, S2 mediates the fusion of viral and cellular membranes thus ensuring viral entry through endocytosis (Jaimes et al., 2020; Ou et al., 2020). Understanding the conformational changes of the protein can help with the development of therapeutics. The receptor-binding domain (RBD) on the S protein is essential to infect host cells. While the RBD within the S1 region varies between CoVs, a common property that occurs is the cleavage of the S protein (Jaimes et al., 2020). It was shown that the SARS-CoV-2 RBD has a higher affinity to ACE2 compared to the SARS-CoV and RaTG13 RBD (Li et al., 2020b). However, the affinity of the entire SARS-CoV-2 S protein is similar to the SARS-CoV S protein, suggesting that SARS-CoV-2 RBD is less exposed than the SARS-CoV RBD (Liu et al., 2020b; Shang et al., 2020b; Wrobel et al., 2020). The high binding affinity of the RBD to ACE2 and the hidden RBD in the S protein may allow SARS-CoV-2 to maintain efficient cell entry while simultaneously evading immune surveillance (Shang et al., 2020a).

The mechanism which SARS-CoV-2 uses for cleavage is similar to that of other CoVs; However, recent studies demonstrated a furin pre-activation can facilitate the entry into cells, especially with those with low expressions of TMPRSS2 and/or lysosomal cathepsins (Shang et al., 2020a). After the RBD in S1 attaches to the host ACE2 receptor, the cell entry of CoVs is mostly regulated by acid-dependent proteolytic cleavage of the S protein by cathepsin, transmembrane protease serine 2 (TMPRRS2), FURIN or other proteases (Astuti and Ysrafil, 2020; Rabi et al., 2020; Shang et al., 2020a). This cleavage must occur at two sites within the S2 subunit before gaining access to the host cytosol. The first cleavage separates the RBD and the fusion domains of the S protein and the second exposes the fusion peptide. The peptide is then inserted into the plasma membrane, combining both host and viral membranes. An additional study demonstrated that SARS-CoV-2 enters the cell through endocytosis, and that blocking phosphatidylinositol 3-phosphate 5-kinase (PIKfyve), an enzyme that synthesizes the phosphoinositides that are needed for endocytosis, significantly reduced SARS-CoV-2 from cell entry (Ou et al., 2020). They also demonstrated that blocking a downstream effector of the phosphoinositides, the two-pore channel subtype 2 (TPC2) also decreased cell entry. This data provides insights into additional inhibition targets to reduce cell entry. Once the viral and cellular membranes have fused, the viral genome is then released into the cytoplasm (Astuti and Ysrafil, 2020; Rabi et al., 2020).

After the viral genomic RNA has entered the host cell, the next step is the translation of the replicase gene. The replicase gene encodes two large ORF, rep1a and rep1b which expresses two co-terminal polyproteins, pp1a and pp1ab. During replication, the RTC drives the production of full-length negative-sense RNA copies of the viral genome. The negative-sense RNA is later used as templates for the production of full-length (+) RNA copies. During transcription, a nested set of sub-genomic RNAs (sgRNAs) is produced in a manner of discontinuous transcription (fragmented transcription). The protein products of rep1a and rep1b contain nonstructural proteins (NSPs) 1-11 and 1-16, respectively. The NSPs are necessary for forming the replication organelle, RTC, thus providing an optimal environment for RNA synthesis (Du Toit, 2020). After replication and sub-genomic RNA synthesis, structural proteins and nucleocapsids are translated and inserted into the endoplasmic reticulum (ER). While these proteins move to the endoplasmic reticulum-Golgi intermediate compartment (ERGIC), the RNA genome, encapsidated by the N protein, buds into the membranes of ERGIC (Alanagreh et al., 2020). When the structural proteins and RNA meet in the ERGIC, mature virions are formed. Both E and M proteins are essential for virion assembly, however, the expression of E is far less than M. The M protein is responsible for determining the shape of the virus and also helps with nucleocapsids, thus promoting the completion of the viral assembly by stabilizing the N protein and the RNA complex. While S protein may be incorporated at this step, it is not critical for assembly. Once the assembly is completed, the virions are transported to the cell surface in vesicles and released by exocytosis. (Fehr and Perlman, 2015).

## Epidemiology/Transmission

While there is a myriad of CoVs that are known to cause the common cold in humans, the last three CoV outbreaks of the century have caused an increase in ARDS. The first pneumonia outbreak caused by SARS-CoV originated from Guangdong province, China, and led to an increase in severe acute respiratory syndrome (SARS) (Cherry and Krogstad, 2004). In 2012, another novel CoV outbreak occurred in Saudi Arabia and was given the name Middle East Respiratory Syndrome-CoV (MERS-CoV) (De Wit et al., 2016).

**Figure 2:**
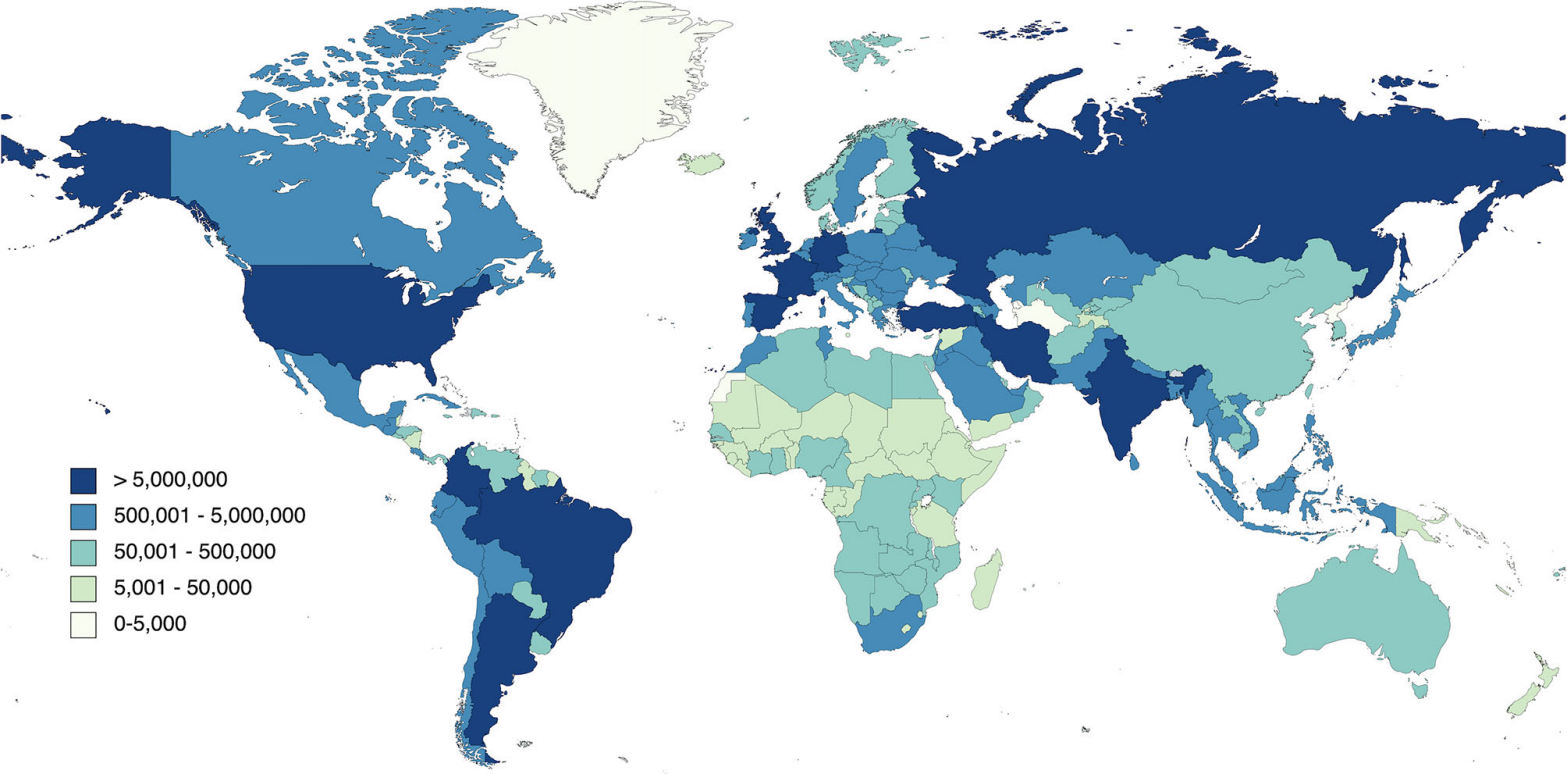
The rapid spread of COVID-19 worldwide.

Years after the incidence of SARS and MERS, the first pneumonia cases with an unknown origin were documented in Wuhan China late in December of 2019. The earliest reported case dated back to November 17, and is believed to be a 55-year-old man from Hubei province (Lu, 2020). Thus, the virus likely crossed the animal to human barrier in late 2019. SARS-CoV-2 is predicted to have a zoonotic origin since a majority of the first patients had connections to the wet animal market in Wuhan City where live animals are sold (Zhou et al., 2020). As of November 2021, there have been over 215 million cases worldwide, including about 5 million deaths of COVID-19 in 222 countries and territories (**Figure 2**) (WHO, 2020b). While individuals of all ages are at risk for infection and severe disease, people over the age of 50 and with chronic medical conditions are more susceptible and likely to develop severe forms of COVID-19. Meta-analysis from a study discovered that patients with hypertension, cardiovascular diseases, diabetes, smoking habits, chronic obstructive pulmonary disease (COPD), and chronic kidney disease exhibited severe COVID-19 symptoms marking these as the most frequent underlying conditions reported in hospitalized patients (Bulut and Kato, 2020; Shi et al., 2020). Other studies have also demonstrated that active cigarette smoking and COPD upregulate ACE-2 expression in the lower airways, which in part may explain the increased risk of severe COVID-19 in these populations. This highlights the importance of increased surveillance of these risk subgroups for the prevention and rapid diagnosis of this disease (Leung et al., 2020).

The estimated median incubation period of SARS-CoV-2 is 5.1 days, and patients are expected to develop symptoms within 12 days of infection (Lauer et al., 2020). These aspects of the infection support current quarantine protocols and active monitoring proposals; however, the quarantine time should be extended to about 14 days for those exposed or associated with high-risk scenarios (Lauer et al., 2020). While studies have suggested that COVID-19 may be airborne, the main routes of transmission are respiratory droplets and close contact transmission from person to person. Person-to-person transmission of the virus can be facilitated through coughs, sneezes, and exhales (Prompetchara, 2020). The basic Reproductive Number (R_0_) refers to the average number of secondary infections generated by an infected individual over the course of its infectious period. If R_0_ >1, then the number of infected individuals multiplies, and transmission increases. For SARS-CoV-2 the R_0_ is estimated to be 3.28; however, discrepancies such as numerous biologic-, socio-behavioral, and environmental factors exist depending on the models used for quantification (Sun et al., 2012). The R_0_ range for COVID-19 is expected to be between 2-3 (Li et al., 2020a; Liu et al., 2020c) compared to SARS (2.3-3.7) (Bauch et al., 2005; Sun et al., 2012). More interestingly, the R_0_ for the delta variant of SARS-CoV-2 is 5.08, much higher than the ancestral strain of SARS-CoV-2 as well as other viral infections such as SARS, MERS, smallpox, Ebola, seasonal and pandemic influenza [(Liu and Rocklov, 2021) J of Travel Meda]. Control measures, such as the implementation of rapid diagnosis and effective isolation of patients, aim to reduce the number to less than 1 (Li et al., 2020a).

## Clinical Characteristics and Management of COVID-19

In the pre-SARS era, human coronaviruses were known to cause mild respiratory diseases only; however, this changed after the SARS outbreak in 2002 (Fung et al., 2020). The first known highly pathogenic coronaviruses, SARS- CoV and MERS- CoV, caused severe respiratory illnesses such as acute respiratory distress syndrome (ARDS), while the other four human coronaviruses (HCoV- NL63, HCoV-229E, HCoV- OC43, and HKU1) induced mild upper respiratory diseases in the elder population, children, and immunocompetent patients (Cui et al., 2019; Ahn et al., 2020).

SARS-COV-2 predominantly infects the lower respiratory tract and causes pneumonia in humans (Chen et al., 2020b). The most susceptible cells to SARS-CoV-2 infection are those with increased expression of ACE2 such as goblet and ciliated cells in the nose, type two pneumocytes in the lung, adsorptive enterocytes in the gut, endothelial cells in circulation, cardiac myocytes in the heart, and olfactory nerves in the central nervous system (CNS) (Hamming et al., 2004). COVID-19 patients either stay asymptomatic or experience mild, moderate to severe symptoms. Common clinical findings of COVID-19 include leukopenia and lymphopenia, as well as abnormalities that include elevated levels of aminotransferases, C-reactive protein, D-dimer, ferritin, and lactate dehydrogenase (Thell et al., 2021). Symptoms are more severe in older populations with pre-existing medical conditions- the most common being cardiovascular disease, diabetes, chronic kidney disease, and chronic lung disease (Guo et al., 2020). Moreover, patients suffering severe COVID-19 with comorbidities were also shown to express high levels of ACE2 in lung tissues, suggesting that these patients are more susceptible to infection (Pinto et al., 2020). Other risk factors include allergic diseases, asthma, and chronic obstructive pulmonary disease (COPD) (Ahn et al., 2020). The majority of COVID-19 patients suffer from mild flu-like symptoms including fever, dry cough, fatigue, expectoration, dyspnea, headache or dizziness, diarrhea, nausea, and vomiting. (Li et al., 2020a). The risk of viral pneumonia increases with age. A small percentage of infected individuals develop severe complications including ARDS, respiratory failure, liver injury, acute myocardial injury, acute kidney injury, septic shock, and even multiple organ failure (Shi et al., 2020). Death results through alveolar injury, impeding airway capacity, and multiorgan failure both of which are associated with hyperproduction of cytokines. Emerging data suggest that COVID-19 patients may also develop extrapulmonary manifestations and complications such as cardiac, dermatologic, hematological, hepatic, neurological, renal, and other complications (Agarwal et al., 2020; Henry et al., 2020; Liu et al., 2020b; Madjid et al., 2020; Sachdeva et al., 2020). In the early stages of COVID-19, studies have shown an increase in mucus-based barriers that influence O2 diffusion without altering CO2 diffusion, suggesting that patients can maintain relative physiological homeostasis without presenting overt symptoms, a clinical feature known as silent hypoxia (Huang et al., 2020; Liu et al., 2020d).

There are currently two types of tests for COVID-19: molecular and antibody tests. Molecular tests are used to confirm active infection and consists of molecular tests, such as RT-PCR and antigen tests. RT-PCR detects viral genetic material and viral proteins such as the S protein and is often more sensitive than antibody tests, however, these tests do not measure infectivity. Researchers have also developed a test using RT-PCR to detect negative cases of SARS-CoV-2 using the SYBR Green methodology, a dye that binds the minor groove of double-stranded DNA, increasing the fluorescence intensity (Marinowic et al., 2021). Antibody tests are used to determine previous exposure to the virus by detecting levels of antibody specific for the virus but do not confirm active infection, therefore, are not used for diagnosis. While molecular tests can confirm active infection, they cannot confirm whether the patient is infectious or not. This highlights the need for the development of an assay to determine the infectious state of the patient to prevent further spread.

## Treatment

The pathogenesis of COVID-19 is thought to be driven by two main processes. Early on in the infection, the disease is driven by the adverse replication of SARS-CoV-2. After prolonged replication, the disease is driven by the hyperactivation of immune/inflammatory responses to the virus, leading to tissue damage (Kim et al., 2020). To date, the Food and Drug Administration (FDA) has approved Remdesivir for the treatment of COVID-19 for adults and pediatric patients and is recommended for use in hospitalized patients who require supplemental oxygen (FDA, 2020). Current therapeutics for hospitalized patients include antiviral treatments that directly target the virus, such as Remdesivir, and a corticosteroid, Dexamethasone. For the earliest stages of infection, anti-SARS-CoV-2 monoclonal antibodies, such as bamlanivimab and casirivimab plus imdevimab may benefit patients who are at high risk for disease progression and are available through Emergency Use Authorizations recommended for treatments: Lopinavir/Ritonavir, Ivermectin, and interleukin-6 inhibitors (Elavarasi et al., 2020). Convalescent plasma for hospitalized patients has been issued an Emergency Use Authorization (EUA) by the FDA for COVID-19 treatment; however, there is insufficient data to recommend either for or against the use of convalescent plasma (Health, 2020; WHO, 2020a). Another potential therapeutic for COVID-19 is REGN-COV-2, which is a combination of two monoclonal antibodies (REGN10933 and REGN10987) by Regeneron designed to block the infectivity of SARS-CoV-2 [COVID-19 Treatment Guidelines Panel. Coronavirus Disease 2019 (COVID-19) Treatment Guidelines, 2020; Baum et al., 2020; Brouwer et al., 2020].

Although none have been FDA-approved, many potential therapeutics are currently being investigated for the treatment of COVID-19. For example, Dalbavancin was found to block the interaction between ACE2 and SARS-CoV-2 S protein in both mouse and non-human primate animal models (Wang et al., 2021b). Other studies have found that a human neutralizing antibody cocktail conferred protection against SARS-CoV-2 and SARS-CoV *in vitro*, while other studies demonstrated protection against SARS-CoV-2 using two monoclonal antibodies in mice (Yao et al., 2021; Zhang et al., 2021).

Given the inter-individual variations of COVID-19, clinical manifestations, the efficacy of one treatment regimen is neither enough nor sufficient to treat patients covering a broad spectrum of the disease. The underlying heterogeneity of many diseases, including COVID-19, suggests that strategies for treating an individual with a disease, and possibly monitoring or preventing that disease, must be ‘personalized’ to that individual’s unique biochemical, physiological, environmental exposure, and behavioral profile. The availability of modern biomedical technologies such as DNA sequencing, genomics, and proteomics to access an individual’s susceptibility to disease is now being recognized as a more efficient method for treatment.

## Personalized Medicine

The focus of personalized or precision medicine is to explore how treatment or prevention approaches can be developed based on a combination of factors such as genetic environmental, and social factors. Advancements in computational tools such as DNA sequencing, genomics, and other ‘omic’ technologies have allowed us to identify health and disease determinants as well as develop individualized and population-level interventions to treat and prevent human disease (Goetz and Schork, 2018). The subclassification of infectious disease requires consideration of both host and pathogen. Because of the considerable variation of COVID-19 disease, understanding inter-individual heterogeneity in susceptibility to disease and severity of illness is critical.

The heterogeneity of COVID-19 is a result of multiple factors including different variants of SARS-CoV-2, host variability, and differential immune responses. High dimensional studies in humans and animals have led to a better understanding of immune responses in COVID-19, with human studies developing a better understanding of the late-phase disease, and animal studies on the dynamics of disease. Because multiple distinct disease phenotypes and marked differential responses to immunosuppressive therapy exist in patients with COVID-19, additional studies have also focused on genome-wide association analysis (GWAS), transcriptome-wide association studies (TWAS), and Mendelian randomization to further identify and characterize the potential genetic factors involved in the development of COVID-19 (Ma et al., 2021; Pairo-Castineira et al., 2021).

Using genome-wide studies, researchers have found that critical illness in COVID-19 is related to at least two biological mechanisms: innate antiviral defense, such as early type 1 interferon (IFN) signaling and host-driven inflammatory lung injury. These studies have found that low expression of IFNAR2, or high expression of TYK2, are associated with life-threatening disease and high expression of the monocyte–macrophage chemotactic receptor CCR2 in lung tissue is associated with severe COVID-19 (Dorward et al., 2021; Ma et al., 2021; Pairo-Castineira et al., 2021).

The exact reason for exaggerated and misdirected host immune responses and varied clinical manifestations in COVID-19 is a continued investigation. Given the unpredictability of severe disease development, early administration of type 1 IFN treatment is one of many probable treatments that may be beneficial in patients with severe COVID-19 since there is an early loss-of-function phenotype in IFN signaling (Lee and Shin, 2020; Pairo-Castineira et al., 2021). Additionally, because COVID-19 shares similarities with autoimmune diseases several studies have also identified the production of autoantibodies in patients with COVID-19 (Hejrati et al., 2020; Wang et al., 2021a). A study investigating the production of autoantibodies in patients infected with COVID-19 discovered a high prevalence of autoantibodies against immunomodulatory proteins including cytokines, chemokines, complement components and cell-surface proteins, all of which are necessary to elicit an appropriate immune response (Wang et al., 2021a). An independent study reported inborn errors of type I IFN immunity in patients with life-threatening COVID-19 (Zhang et al., 2020a). Interestingly, another study found a correlation between life-threatening COVID-19 and neutralizing auto-antibodies against type I IFNs, having a more pronounced excess within the male population, adding to the ongoing research of increased susceptibility in men (Bastard et al., 2020). These results suggest a pathological role for extracellular or secreted autoantibodies and disruption of innate type I IFN response in COVID-19, having diverse effects on immune functionality and associations with clinical outcomes, especially within the male population.

Epidemiological data have shown differential impact of SARS-CoV-2 infection between males and females. While both sexes are similarly infected, the outcome favors the female population, thus suggesting a potential role of sex biology during infection. Because low testosterone levels have been associated with increased metabolic risk and systemic inflammation, many studies investigating genetic polymorphisms that predispose some men to developing more severe disease have shown correlations between severe COVID-19 and men, particularly androgen receptor (AR) and testosterone levels (Mohamad et al., 2019; Baldassarri et al., 2021). One study investigating genetic variability due to poly-amino acid repeats using supervised Machine Learning (Baldassarri et al., 2021) compared the genotypes of patients with extreme clinical manifestations (asymptomatic vs. severe), and found an association between the poly-glutamine (Q) repeat number of the AR gene, serum testosterone concentrations, and COVID-19 outcome in male patients. They concluded that the inability of the endocrine feedback to overcome AR signaling defects through the increase of testosterone levels during the infection leads to the polyQ tract becoming dominant to serum testosterone levels for the clinical outcome. These data are in line with another study that demonstrated lower baseline testosterone levels predict poor prognosis and mortality in SARS-CoV-2 infected men (Rastrelli et al., 2021).

The COVID-19 severity outcome in patients is driven by many factors, which may be the reason why patients respond differently to treatment. Studies on the development of precision medicine are too costly at the moment; however, these studies have established molecular mechanisms, including pathways and comorbidities in people that can be key determinants to develop responsive treatments in susceptible individuals and populations paving way for the end goal of precision medicine: targeted therapeutics to prevent severe manifestations of disease, especially in COVID-19.

## Discussion

COVID-19 has been the greatest large-scale global pandemic caused by a CoV yet, with over 215 million confirmed cases and approximately 5 million deaths, as of November 2021. The emergence of pandemics of animal origin is a growing threat, accounting for about two-thirds of human infectious diseases. The most devastating pandemics in history are the Justinian Plague (541-542 AD), the Black Death (Europe, 1347), yellow fever (South America, sixteenth century), the Spanish Flu (1918), and modern-day pandemics such as HIV/AIDS, and the highly pathogenic H5N1 flu, all of which are animal origin (Machalaba et al., 2015). It is therefore of no surprise that SARS-CoV-2 crossed the species barrier and caused a global pandemic, and it is very likely that SARS-CoV-2 will not be the last to cause catastrophe in the human population.

Before identifying effective therapeutics or developing a vaccine against infection, it is crucial to have a thorough understanding of the emerging pathogen. During the early phase of the pandemic, researchers across the globe shifted their research focus to gain more information on SARS-CoV-2. Within a year, the first vaccine doses were being distributed across the world. With ongoing research, new vaccine platforms have been investigated and therapeutics against COVID-19 are grossly improving. From determining the cause of the pneumonia outbreak in Wuhan China to distributing vaccines worldwide in a little over a year, our greatest efforts to end the global pandemic have required extensive support from all personnel. Moreover, it is also critical that we continue implementing safety measures for this ongoing pandemic. While we may have the technology and resources to continue fighting against SARS-CoV-2, it is important to note that there are still many unknowns to uncover to successfully eliminate the pathogen. As we get closer to defeating COVID-19, we continue to learn that there are many strategies the virus may have to combat our efforts, such as mutating to becoming more transmittable like the delta variant or making out vaccines less effective.

Although many contemporary technologies in scientific research and medicine were major contributors to bringing the pandemic closer to an end, many lives worldwide were astronomically affected. While scientific research led to the rapid discovery of the etiological agent of COVID-19 and the development of vaccines against COVID-19 in less than a year, the next global pandemic could be avoided if we invest more in the preparedness for future health emergencies.

A greater understanding of clinical manifestations and varied immune responses across all patient cohorts by mapping the genetic architecture of disease using high throughput studies like genome-wide and transcriptome-wide association studies and Mendelian randomization, clinicians can direct a more effective treatment to their patients. Given the heterogeneity of many diseases, especially COVID-19, personalized or precision medicine may be one of the more effective treatments for all patients.

## Author Contributions

RE, DK, and DS wrote the draft. RE prepared the illustrations. All authors contributed to the discussion and final editing. All authors contributed to the article and approved the submitted version.

## Funding

U42OD010442 (NIH Office of the Director), P51OD011133 (NIH Office of the Director), NIH award # R01AI123780, R01AI134236 to DK and COVID-19 research grant from San Antonio Medical Foundation to DS.

## Conflict of Interest

The authors declare that the research was conducted in the absence of any commercial or financial relationships that could be construed as a potential conflict of interest.

## Publisher’s Note

All claims expressed in this article are solely those of the authors and do not necessarily represent those of their affiliated organizations, or those of the publisher, the editors and the reviewers. Any product that may be evaluated in this article, or claim that may be made by its manufacturer, is not guaranteed or endorsed by the publisher.
